# Tetranucleotide usage highlights genomic heterogeneity among mycobacteriophages

**DOI:** 10.12688/f1000research.6077.2

**Published:** 2015-10-30

**Authors:** Benjamin Siranosian, Sudheesha Perera, Edward Williams, Chen Ye, Christopher de Graffenried, Peter Shank

**Affiliations:** 1 Center for Computational Molecular Biology, Brown University, Providence, RI, 02912, USA; 2 Division of Biology and Medicine, Brown University, Providence, RI, 02912, USA; 3 Department of Molecular Microbiology and Immunology, Brown University, Providence, RI, 02912, USA

**Keywords:** mycobacteriophages, computed, tetranucleotide, usage, deviation, genome, sequences

## Abstract

**Background**

The genomic sequences of mycobacteriophages, phages infecting mycobacterial hosts, are diverse and mosaic. Mycobacteriophages often share little nucleotide similarity, but most of them have been grouped into lettered clusters and further into subclusters. Traditionally, mycobacteriophage genomes are analyzed based on sequence alignment or knowledge of gene content. However, these approaches are computationally expensive and can be ineffective for significantly diverged sequences. As an alternative to alignment-based genome analysis, we evaluated tetranucleotide usage in mycobacteriophage genomes. These methods make it easier to characterize features of the mycobacteriophage population at many scales.

**Description**

We computed tetranucleotide usage deviation (TUD), the ratio of observed counts of 4-mers in a genome to the expected count under a null model. TUD values are comparable between members of a phage subcluster and distinct between subclusters. With few exceptions, neighbor joining phylogenetic trees and hierarchical clustering dendrograms constructed using TUD values place phages in a monophyletic clade with members of the same subcluster. Regions in a genome with exceptional TUD values can point to interesting features of genomic architecture. Finally, we found that subcluster B3 mycobacteriophages contain significantly overrepresented 4-mers and 6-mers that are atypical of phage genomes.

**Conclusions**

Statistics based on tetranucleotide usage support established clustering of mycobacteriophages and can uncover interesting relationships within and between sequenced phage genomes. These methods are efficient to compute and do not require sequence alignment or knowledge of gene content. The code to download mycobacteriophage genome sequences and reproduce our analysis is freely available at https://github.com/bsiranosian/tango_final.

## Introduction

Mycobacteriophages, phages infecting mycobacterial hosts, are a subset of the estimated 10 ^31^ phage particles present globally. Mycobacteriophages infect a number of bacterial hosts from the genus *Mycobacterium*, and they are broadly classified into *Siphoviridae* and *Myoviridae*. Mycobacteriophages are present in both land and aquatic environments and play a large ecological role in the turnover and evolution of bacteria ( Bohannan & Lenski, 2000; Chibani-Chennoufi *et al.*, 2004; Hendrix, 2002). The recent rise of antimicrobial-resistant pathogenic bacteria has renewed interest in mycobacteriophages and the potential for phage therapy of *Mycobacterium tuberculosis* infections. Although *in vivo* experiments have not yet yielded promising clinical results, mycobacteriophages are still powerful diagnostic tools for the investigation of mycobacterial pathogenesis ( Danelishvili, 2006; Hatfull, 2014; McNerney, 1999).

The genomic sequences of mycobacteriophages are mosaic and diverse. As of April 2014, 663 distinct mycobacteriophage genomes were available on the database PhagesDB.org; most were isolated on *Mycobacterium smegmatis* MC ^2^155. Global Guanine + Cytosine (GC) content ranges from 50.3% to 70% (mean of 63.9%), and genome lengths range from 41kb to 165kb (mean of 67kb). In total, more than 50,000 distinct genes are found within the population. The majority of these genes are of unknown function and do not have homologs in other types of phages or bacteria ( Hatfull *et al.*, 2010). However, many genes are shared between closely related mycobacteriophages. Similar genes have been grouped into almost 4,000 phamilies (or phams, a play on gene families) based on shared amino acid sequence. Phams have been used to investigate horizontal gene transfer within the mycobacteriophage population and to create phylogenetic trees.

Despite the high levels of diversity, mycobacteriophages can be grouped into distinct clusters based on their morphologic and genetic features. Some clusters are large and further divided into subclusters (cluster A, for example, with 11 subclusters and 246 members), while other are small and undivided (cluster S with two members and no subclusters). Some phages have no nearest neighbor to establish a cluster and are classified as singletons. Clusters are defined using four methods: dot-plot comparisons, pairwise average nucleotide identities, pairwise genome map comparisons and gene content analysis ( Hatfull *et al.*, 2010). However, it should be noted that the clustering scheme proposed for mycobacteriophages mainly serves to identify similarities in genome architecture. This clustering scheme, and our proposed methods of grouping based on tetranucleotide usage described below, are not true taxonomic representations of the mycobacteriophage population. Extensive horizontal gene transfer prevents accurate reconstruction of evolutionary history from purely phylogenetic information ( Lawrence *et al.*, 2002).

Methods traditionally used to analyze mycobacteriophage genomes require sequence alignment or genome annotation. These analytical tasks can be effective, but they are not without drawbacks. Alignment-based methods can be biased by the choice of score parameters ( Frith *et al.*, 2010), and genome annotation may require significant manual input, including by-hand verification of automated gene calls before a mycobacteriophage genome is submitted to GenBank. It is especially difficult to build multiple-sequence alignment based phylogenetic trees from mycobacteriophage genomes because phages lack a common genetic element, such as 16S rRNA in bacteria ( Doolittle, 1999). Alignment-free methods avoid many of the disadvantages associated with alignment-based inference. These methods typically use statistics based on the oligonucleotide composition of a sequence and are completely independent of alignment or annotation. Several methods have been developed for different applications; most are covered in the excellent review by Vinga (2007). Alignment-free methods are also less computationally intensive than multiple sequence alignment. While the complexity of sequence alignment algorithms scales at least as fast as the square of the number of sequences (at least O( *n ^2^*) complexity), alignment free methods typically fall below O( *n ^2^*) ( Chan & Ragan, 2013).

Even so, there are drawbacks to alignment-free methods for analyzing genomes, mostly related to the interpretation of statistics in an evolutionary context. It can be difficult to understand how oligonucleotide frequencies are modified in a population over time when selection usually takes place at the level of genes. Oligonucleotide frequencies can also be subject to convergent evolution: if two distantly related phages slowly converge to similar usage frequencies, these methods can give a false indication of common ancestry ( Pride *et al.*, 2003).

Alignment-free methods have been used to study phage and bacterial genomes in a variety of contexts. For example, Pride *et al.* (2006) found tetranucleotide usage to carry a strong phylogenetic signal in bacteriophages and showed that tetranucleotide composition was similar among phages with common hosts. More recently, Ogilvie *et al.* (2013) surveyed metagenomic sequencing datasets using a tetranucleotide usage-based method and discovered several novel *Bacteroidales*-like phages which could not be identified with alignment-based methods. Oligonucleotide composition vectors have also been proposed as a method to root viral phylogenies ( Simmons, 2008).

Statistics based on nucleotide composition in a sliding window can theoretically be used to uncover horizontal gene transfer (HGT), based on the assumption that genomes have self-similar nucleotide composition and outlier regions could represent recent horizontal transfer events ( Lawrence & Ochman, 1997). Guanine + Cytosine (GC) content in a sliding window was first used to look for pathogenicity islands within a genome ( Hacker & Kaper, 2000). More recent methods have used nucleotide composition and Naïve Bayesian classifiers ( Sandberg *et al.*, 2001) or hidden Markov models ( Waack *et al.*, 2006). However, if horizontally transferred segments change in oligonucleotide composition to be more similar to the resident genome, a process known as amelioration, it can obscure truly horizontally transferred segments ( Koski *et al.*, 2001).

The number of sequenced mycobacteriophages has grown immensely in the past few years thanks to the Howard Hughes Medical Institute (HHMI) Science Education Alliance Phage Hunters Advancing Genomics and Evolutionary Science (SEA-PHAGES) course ( Jordan *et al.*, 2014). This program allows first year undergraduate students to isolate and characterize novel mycobacteriophages from the environment. It has also provided excellent opportunities for collaborative projects between undergraduates, resulting in the work presented here Siranosian *et al.* (2015a).

As the number of sequenced mycobacteriophages continues to increase, researchers need new methods to quickly make comparisons at many scales. Alignment-free methods are one possibility: they are independent of sequence alignment or genome annotation, less computationally complex than alignment-based methods and applicable to genomes without a common subsequence. We investigated tetranucleotide usage in mycobacteriophage genomes as an alignment-free alternative to traditional methods for genome comparison. Our findings support what is known about mycobacteriophage biology: phages form identifiable groups and subgroups, known as clusters, but have extensive differences between clusters. Tetranucleotide usage also highlights outliers in the population and can describe unique genomic features. All of the analyses here can be done in minutes on a personal laptop. Tetranucleotide usage is a powerful tool to quickly investigate features of the growing mycobacteriophage population.

## Methods

We obtained the genomic sequences of all 663 sequenced mycobacteriophages publicly available on the website PhagesDB.org as of April 2014. This dataset contains both unpublished genomes and genomes available on GenBank. There is not an easy way to download the mycobacteriophage database in its entirety, so we automated the process with a Python script available in the code accompanying this manuscript.

To compare mycobacteriophage genomes independently of sequence alignment, we investigated the usage of *k*-mers, substrings of DNA of length *k,* in each genome. Given a value for *k*, there are 4 ^*k*^ possible substrings. For example, the 16 possible ways to combine {A, T, C, G} in substrings of length two are {AA, AT, AC, AG, TA, TT, TC, TG, CA, CT, CC, CG, GA, GT, GC, GG}. Different values for *k* are used throughout this paper, but we focus mainly on results from *k*=4 and *k*=6. In the following section, a substring of length *k* is called a word, abbreviated by *W*. Before computing *k*-mer usage, each genome is extended by the reverse complement to account for biases from transcriptional start orientation.

With a chosen value of *k*, we first compute the number of times each substring occurs in the genome. This gives a vector *N* of length 4 ^*k*^, where each entry *N(W)* is the number of times word *W* occurs in the genome sequence. Next, we normalized the *k*-mer frequencies using a zero-order Markov model, which removes biases from the background nucleotide composition and can be effective for analysis of prokaryotic genomes ( Pride *et al.*, 2003; Pride *et al.*, 2006). Normalization accounts for the fact that GC-rich genomes are expected to have more GC-rich *k*-mers simply because of the available nucleotide composition. Dividing the observed counts of *k*-mers by the expected counts highlights *k*-mer usage that can differentiate between mycobacteriophage genomes.

The expected number of a *k*-mer *W* given the background nucleotide distribution is calculated by:

*E*( *W*) = [( *A* ^*a*^ * *T* ^*t*^ * *C* ^*c*^ * *G* ^*g*^) * *N*]

where *A,T,C,G* are the frequency of each nucleotide in the genome, *a,t,c,g* are the number of each nucleotide in the *k*-mer *W,* and *N* is the length of the genome.

The normalized value for a word *W* is calculated by dividing the observed counts by the expected counts. This is the usage deviation vector for a genome, and in the case of *k*=4, tetranucleotide usage deviation (TUD):

*TUD(W) = N(W)/E(W)*

**Figure 1.**
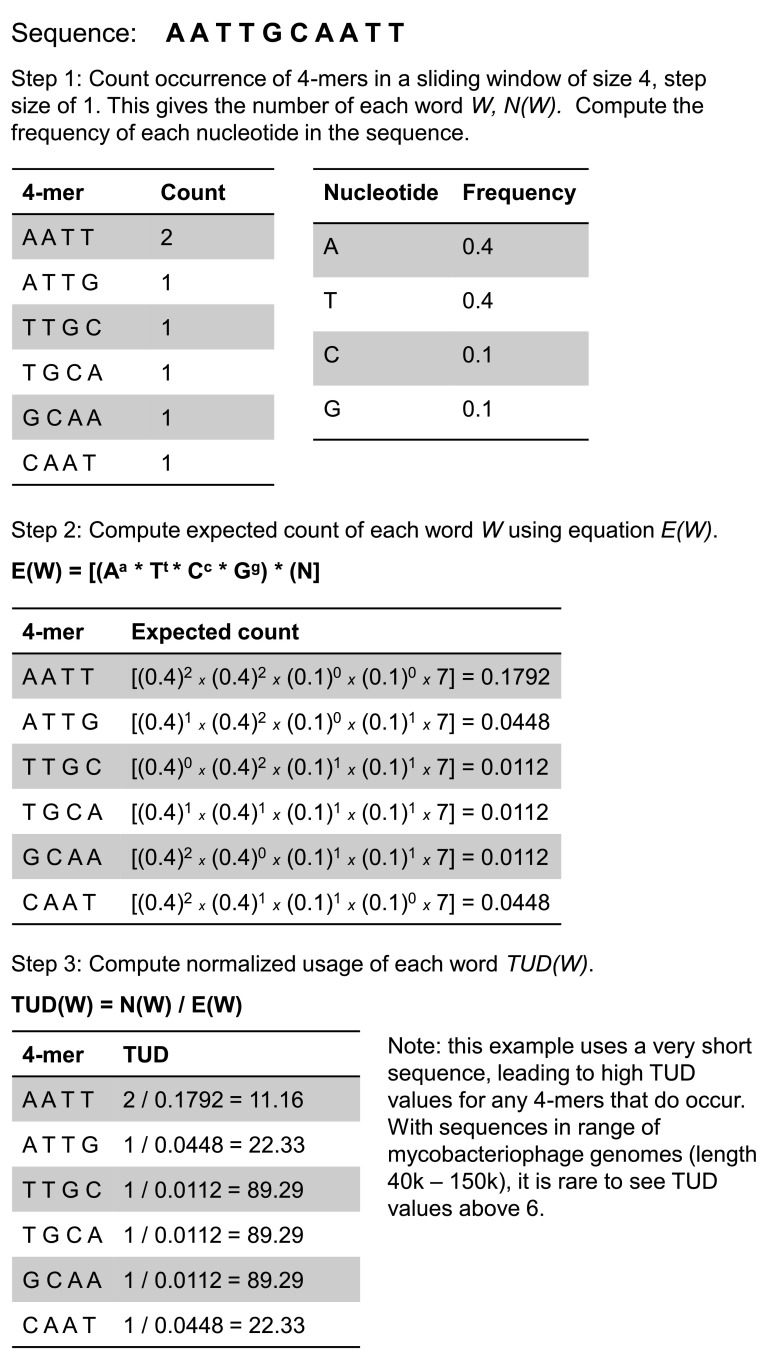
Example of calculating TUD for an input sequence of 10 bases.

An example of calculating TUD values for a short sequence is given in Figure 1. This is equivalent to the “tetranucleotide usage departures from expectation” measure proposed by Pride *et al.* (2003). For a given 4-mer, a TUD value of one corresponds to the expected usage, while a value of two corresponds to usage twice as frequently as expected.

### Data filtering

Phage genomic sequences are extended by the reverse complement before calculation, leading to redundant values for a given tetranucleotide and its reverse complement. One of the redundant tetranucleotides was removed before distance calculations and Principal Components Analysis (PCA). We also removed tetranucleotides that were not present at least once in all phage genomes. Only ATAT and AATT were removed by this filter.

### Comparison of phage genomes

To compare phage genomes in an alignment-free way, we calculated the Euclidean distance between usage deviation vectors. In the case of *k*=4 for a pair of TUD vectors from genomes *x* and *y*:

$$d_{x,y}=\sqrt{\sum_{W=1}^{4^{4}}{\left(TUD_{x}(W)-TUD_{y}(W)\right)}^{2}}$$

Where individual 4-mers are indexed by integers ranging from 1 to 4 ^4^.

Computing pairwise distances between all usage deviation vectors produced a distance matrix used for tree building. For analysis of the subset of 60 phage in Hatful *et al.* (2010), we used the SplitsTree program ( Huson & Bryant, 2006) to construct neighbor joining phylogenetic trees. This was done to facilitate easy comparisons between previously published figures and our alignment-free trees. Hierarchical clustering using the “average” method within the statistical programming language R (version 3.1.0) was used to construct dendrograms for analyzing the entire phage database.

### Principal components analysis

PCA was used to visualize relationships between phage genomes in lower- dimensional space. PCA was done on log-transformed data in R using the ‘prcomp’ function and results were plotted using the ‘ggbiplot’ package.

### Within-genome comparisons

To compare tetranucleotide usage within a phage genome, we used a sliding window of 2000bp (500bp step size). This window size was selected to balance two factors: a short window can detect differences in small regions, while a longer window is necessary to encounter the majority of tetranucleotides. 4-mers were counted and normalized to the nucleotide composition of a given window. A distance matrix was constructed from pairwise Euclidean distances of all windows and used to build heatmaps. Parts of the heatmap where windows overlapped were removed before plotting, leading to the white section along the diagonal in Figure 5.

## Results

### Mycobacteriophage genomes have heterogeneous, yet clustered tetranucleotide usage

**Figure 2.**
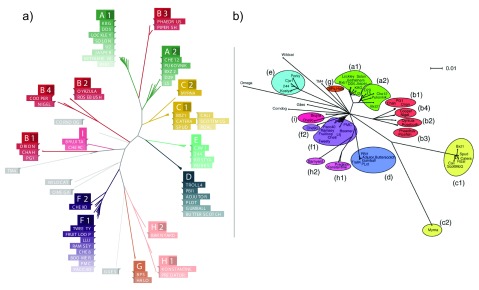
TUD captures similarity within mycobacteriophage subclusters. **a**) Neighbor joining phylogenetic tree constructed from pairwise Euclidean distances between TUD vectors for 60 mycobacteriophage genomes. Phage names are colored based on previously assigned cluster information. **b**) Neighbor joining phylogenetic tree constructed from gene presence data in mycobacteriophage genomes. Reproduced with permission from Figure 3 in Hatful *et al.* (2010). The TUD tree is similar to the alignment-based tree. Phages from the same subcluster form monophyletic clades. In clusters C, F and H, subclusters from the same parent cluster form monophyletic clades.

First, we investigated if TUD reflected relationships described from alignment-based analysis of phage genomes. In particular, does a grouping scheme based on tetranucleotide usage agree with previously assigned phage clusters? To test this hypothesis, we examined a subset of 60 mycobacteriophages first analyzed by Hatfull *et al.* (2010), where the authors propose a clustering scheme based on dot-plot comparisons, pairwise average nucleotide identities, pairwise genome maps and gene content analysis. We calculated the pairwise Euclidean distances between TUD vectors for the subset of 60 phages and used the SplitsTree program ( Huson & Bryant, 2006) to construct a neighbor joining tree ( Figure 2a). Our alignment-free tree has a striking resemblance to the tree from Hatfull *et al.* (2010), which is constructed from similarities in genomic architecture ( Figure 2b). In every case, phages are placed in a monophyletic clade with members of their subcluster.

**Figure 3.**
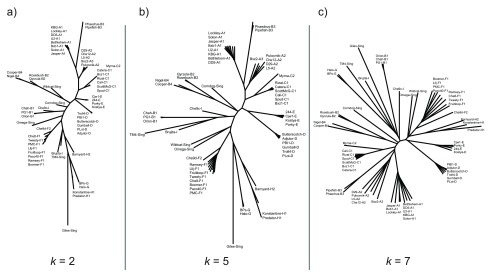
Changing k does not change the structure of the tree. Neighbor joining phylogenetic trees constructed from pairwise Euclidean distances between oligonucleotide usage deviation vectors for 60 mycobacteriophage genomes. Trees from k equal to two, five and seven are shown here. Trees show a high degree of similarity regardless of the k used. Trends observed in the tetranucleotide usage based tree ( Figure 2), such as grouping of subcluster members into monophyletic clades, are conserved in these trees.

Hierarchically grouping phages into clusters and subclusters represents heterogeneity within the mycobacteriophage population. In the alignment-free tree, subclusters from parent clusters C, H and F are placed in a monophyletic clade. However, in some cases, tetranucleotide usage was vastly different between subclusters of a parent cluster. For example, subcluster B3 phages are most similar to cluster A phages in terms of tetranucleotide usage, but they are similar to other cluster B genomes when compared on genetic elements ( Figure 2). We investigate this relationship further in a following section. Importantly, the relationships between the subset of 60 phages are consistent for varying values of *k* ( Figure 3).

**Supplementary Figure 1.**
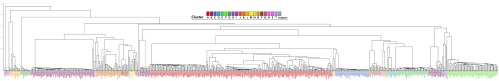
Hierarchical clustering of all 663 phage genomes. Hierarchical clustering dendrogram constructed on pairwise Euclidean distances between all 663 phages in the mycobacteriophage database. In almost every case, phages are placed in a monophyletic clade with members of their subcluster, highlighting the concordance between alignment-based and alignment-free methods for comparison for these genomes. Some clusters (F, C, D, M and L) form monophyletic clades, while others (B, for example) are grouped in different parts of the dendrogram. A larger version of this figure can be downloaded from
Click here for additional data file..

Hundreds of mycobacteriophages have been sequenced in the past few years, bringing the total to 663 genomes ( PhagesDB.org as of April 2014), 21 clusters and 48 subclusters. We next examined TUD patterns in the entire database to see if the relationships observed for the subset of 60 phages were conserved. We used hierarchical clustering within R to analyze this larger dataset (see Methods). As observed for the subset of 60, almost all phages are grouped closely with members of their subcluster. Subclusters of cluster F, C, D, M and L form a monophyletic clade ( Supplementary Figure 1). The relationships for cluster B genomes are also conserved – genomes within a given B subcluster are similar, but the subclusters themselves are different and placed in separate sections of the dendrogram.

### Principal components analysis captures variation in tetranucleotide usage

**Figure 4.**
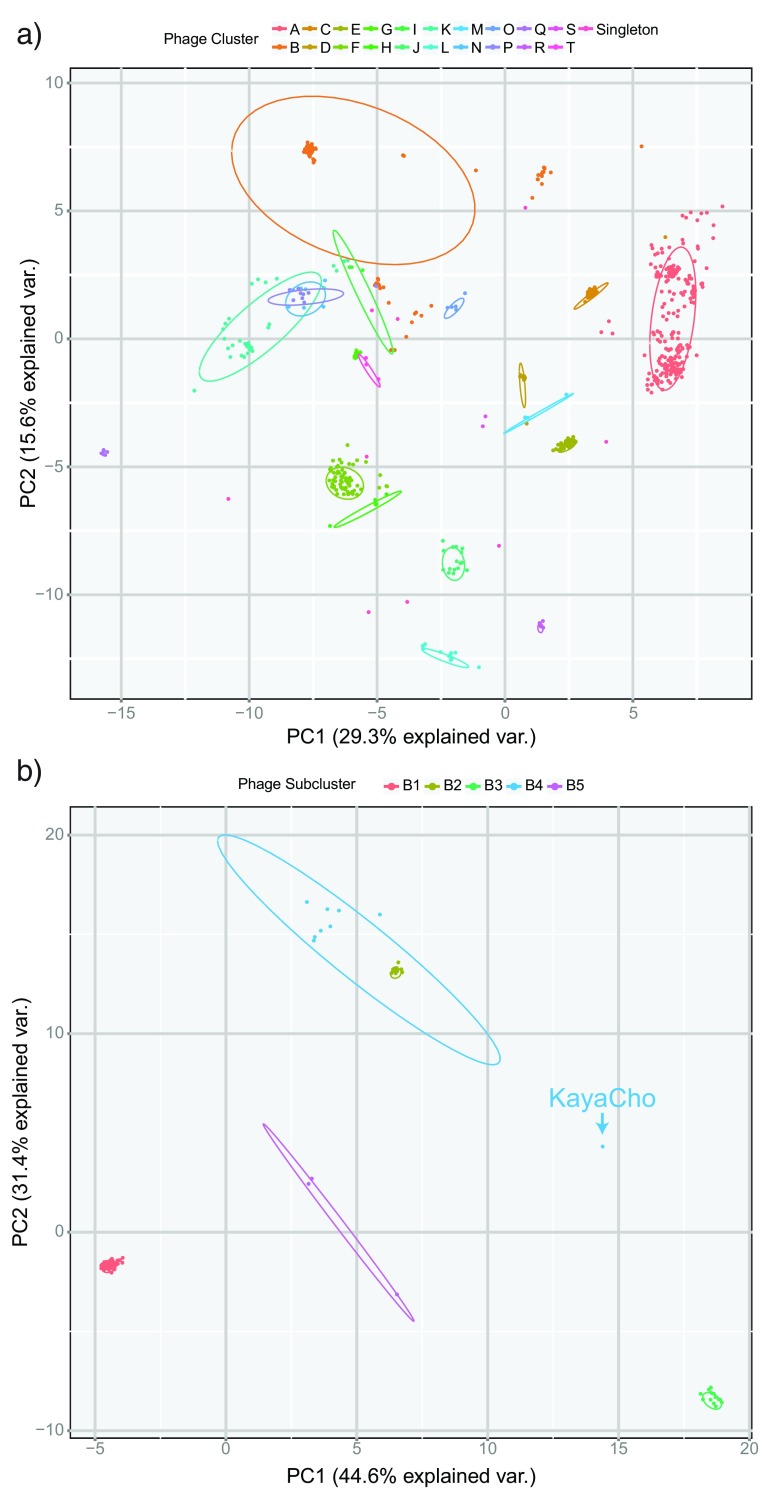
PCA differentiates between clusters and subclusters. **a**) Principal components analysis of all 663 mycobacteriophage genomes. Individual clusters of phages are well separated by PC1 and PC2 in most cases. Further separation can be achieved by incorporating additional principal components. **b**) Principal components analysis of cluster B phages. Individual subclusters are well separated. The outlier in B4 is KayaCho, a phage with different tetranucleotide usage but similar genome architecture when compared with other B4 phages.

We further investigated the ability of TUD to differentiate between predetermined phage clusters using PCA. PCA is useful for visualizing TUD, a 256-dimensional vector, in intuitive 2D space. PCA was applied to log-transformed TUD vectors for all 663 genomes. The first three principal components captured 29.3%, 15.6% and 12.9% of the variance, respectively. Comparing PC1 and PC2 highlighted groups of phage that corresponded well with assigned clusters ( Figure 4a). Clusters that were similar in PC1/PC2 space could be separated further by including additional PCs.

PCA was also useful to compare phages within a single cluster. When comparing cluster B phages, the first three components captured 44.6%, 31.4% and 10.3% of the variance present, respectively. Phage subclusters typically group tightly with each other in PC-space, which makes it easy to detect outliers in terms of TUD. A single member of B4, KayaCho, is placed far from the other genomes of that subcluster ( Figure 4b). This indicates that KayaCho is dissimilar from other B4 phages, a finding that is supported through other methods of comparison. For example, KayaCho has a similar global genome architecture to other members of B4, but pairwise nucleotide identity is low in relation to other comparisons within the subcluster. TUD provides a quick and alignment-free way to detect genomes that are outliers within a subcluster.

### Mycobacteriophage genomes have self-similar tetranucleotide usage, but some regions are outliers

Mycobacteriophage genomes are mosaic and heavily influenced by horizontal gene transfer (HGT) ( Pedulla *et al.*, 2003). We looked for sections within a phage genome that stood out in TUD as potential candidates for HGT events. Tetranucleotide usage was calculated in a 2000bp window with a 500bp step size. Heatmaps of pairwise Euclidean distances between all windows were plotted.

**Figure 5.**
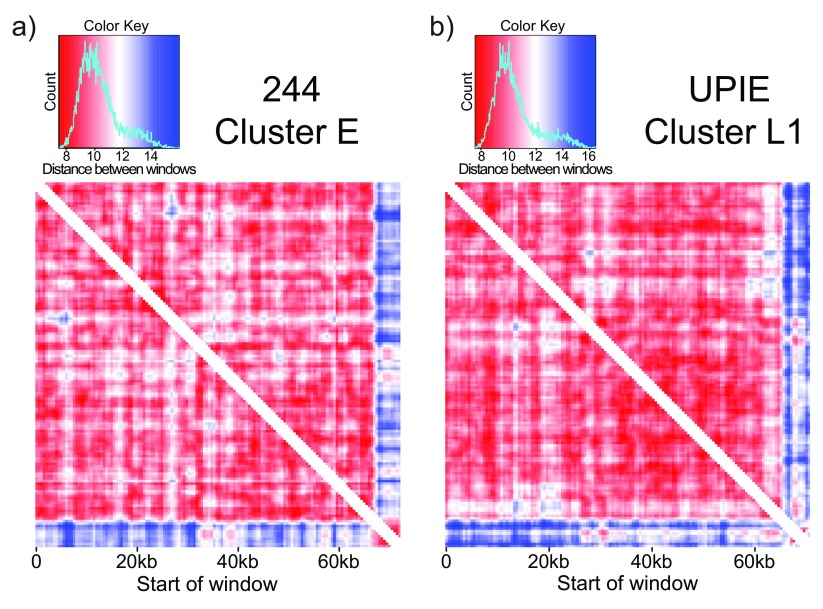
TUD highlights putative horizontally transferred segments. Comparing tetranucleotide usage in a sliding window (2000bp window, 500bp step size) across phage genomes. Each entry in the heatmap is the Euclidean distance between windows. **a**) 244, a cluster E phage, is relatively self-similar with low distance values (red) between most windows. The last 5kb of the genome is an exception: it is self-similar but different than the rest of the genome. This signature is not driven by repetitive sequences, and represents a putative HGT event. **b**) UPIE, a cluster L1 phage, also has a self-similar signature at the end of the genome. However, the difference in TUD in this window is driven by two cluster of repetitive k-mers ( Figure 6).

Observation of these heatmaps revealed several interesting features. The last 5kb of cluster E phage “244” is self-similar, but different than the rest of the genome in terms of TUD ( Figure 5a). This self-similar segment is present with >97% nucleotide identity in all cluster E phage and could represent a HGT event from a different phage cluster or organism. To search for potential transfer sources of this segment, we compared TUD in the region with other mycobacteriophages and searched for nucleotide similarity with BLAST (nr/nt database, blastn algorithm) ( Altschul *et al.*, 1997). However, we were unable to find regions of considerable homology with either method.

**Figure 6.**
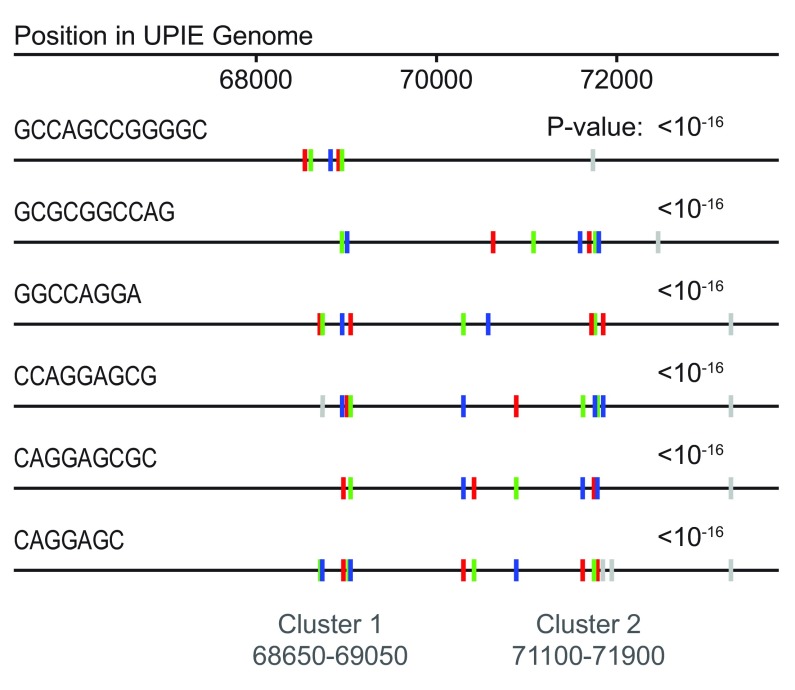
L1 phages contain two clusters of repetitive k-mers. Two clusters of GC-rich repetitive sequences at the end of the genome of UPIE (cluster L1). The repetitive sequences drive the differences in TUD and correspond with the self-similar yet genome-different sections in the within-genome heatmap ( Figure 5). This image was reconstructed from the output of Repfind ( Betley *et al.*, 2002).

Cluster L1 phages contain two small self-similar yet genome-different regions at the end of the genome ( Figure 5b). We examined the genome of “UPIE” with the Repfind program ( Betley *et al.*, 2002) to search for repetitive sequences that could be driving the change in TUD. There are two blocks of repetitive GC-rich *k*-mers, from 68650-69050bp and 71100-71900bp, which match the regions in the heatmap ( Figure 6). As the sliding window moves through each of these blocks, the TUD signal becomes dominated by the repetitive sequence and makes the regions appear self-similar yet genome different. The repetitive features don’t preclude the possibility of HGT in the region, but they do likely obscure a HGT signal carried by TUD. We found other self-similar yet genome-different repetitive regions in phages from clusters F1, H and O. Although the regions highlighted here have variations in GC content, TUD removes biases from the nucleotide composition using a zero-order Markov model (see Methods). Differences in TUD are not a result of variations in the underlying GC content.

### B3 phages contain overrepresented 4-mers and 6-mers

Finally, we examined why B3 phages are not placed with other members of cluster B in the hierarchical clustering dendrogram, while most of the other clusters show this relationship. B3 genomes share greater than 60% average nucleotide identity with other members of cluster B. This is comparable with the relationship between B2 and B4 phages, which are placed close to each other in the dendrogram. The difference in TUD is not likely to be driven solely by differences in pairwise nucleotide identity. We investigated the individual *k*-mers making up the TUD vector to examine this relationship further.

**Figure 7.**
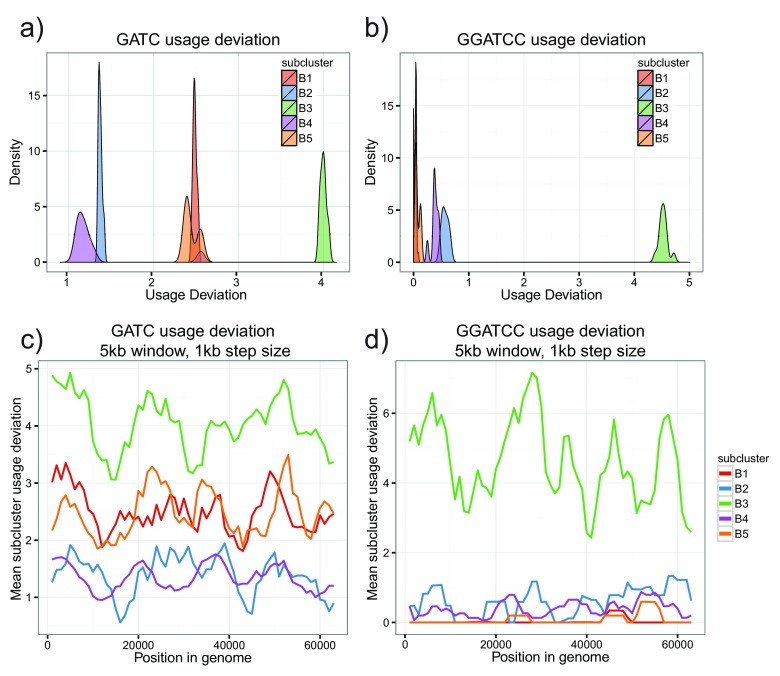
GATC and GGATCC are overrepresented in B3 phages. **a**) Density plot of TUD values for the 4-mer GATC. Individual subclusters form well-defined groups. B3 phages have GATC usage four times what is expected, much higher than other B subclusters. **b**) Repeat of **(a)** with the 6-mer GGATCC. B3 phages use this 6-mer greater than four times what is expected. **c**) GATC usage deviation in a sliding window (5kb, 1kb step size). Each line represents the mean value in the specified subcluster. The increase in GATC usage is genome-wide, indicative of a global change in usage frequency. **d**) Repeat of **(c)** with the 6-mer GGATCC. Increased usage is also genome-wide.

B3 phages used the 4-mer GATC four times more than expected by chance, greater than all other B subclusters ( Figure 7a). The high abundance of GATC could be driven by a global increase in frequency or by discrete regions with very high usage of the 4-mer. To address this point, we compared normalized GATC usage in a sliding window across all cluster B genomes. GATC usage was increased genome-wide in B3 phages, refuting the hypothesis that the deviation was caused by a single genomic region ( Figure 7c). This points to a genome-wide amelioration of GATC usage in cluster B3 genomes. Interestingly, some local peaks and valleys in GATC usage are persistent across all cluster B genomes, even though these genomes are unaligned.

Given the genome-wide increase in B3 GATC usage, it is possible that a higher-order signal could be driving the trend. We searched for highly used 6-mers in B3 phages and found GGATCC had a usage deviation value greater than four, while all other B genomes had a value less than one ( Figure 7b). This increase was also genome-wide ( Figure 7d). GATC and GGATCC are both palindromes, DNA sequences with identical reverse complements. Palindromes are typically underrepresented in bacteriophage and other prokaryotic genomes because they can be parts of recognition sites for restriction enzymes ( Gelfand & Koonin, 1997; Karlin *et al.*, 1992; Sharp, 1986).

GATC is recognized by Dam methylase in *E. coli* ( Marinus & Morris, 1973), but *Mycobacterium* species do not encode Dam methylase ( Hemavathy & Nagaraja, 1995). If B3 phages recently accessed a host with an active Dam methylase, it could lead to a change in GATC frequency. Several restriction enzymes recognize GATC, like *Mgo*I in *Mycobacterium gordonae* ( Shankar & Tyagi, 1993), while others recognize GGATCC, such as *Bam*HI in *Bacillus amyloliquefaciens*. However, the presence of a restriction/modification system in a host would theoretically lead to a decrease in usage of the recognized site. The finding that GATC and GGATCC occur in B3 genomes four times more than expected and significantly more frequently than in all other sequenced mycobacteriophages bears further investigation.

## Discussion

In 2010, there were 60 sequenced mycobacteriophages. There are more than 660 as of April 2014. Alignment-based methods have been used to investigate the mycobacteriophage population, leading to interesting characterizations, such as hierarchical grouping into clusters and subclusters. However, as the number of published genomes continues to grow, there is a need for methods to quickly analyze the entire database of mycobacteriophage sequences.

Throughout this paper, we apply oligonucleotide usage methods to uncover relationships within the population of sequenced mycobacteriophages. These methods allow phage genomes to be compared independently of sequence alignment or genome annotation. The methods for counting *k*-mer usage and normalizing to expected counts are simple to implement and compute. A usage deviation value has a clear interpretation: a value of two corresponds to a *k*-mer occurring twice as frequently as expected in a randomized genome sequence. Usage deviation vectors are also well-suited to distance computation and PCA.

Our findings support what is known about mycobacteriophage biology. Neighbor joining and hierarchical clustering from TUD place closely related phage in well-defined groups that correspond with assigned phage subclusters. In most cases, TUD supports grouping into larger clusters, such as cluster A, where all 246 members form a monophyletic clade in the hierarchical clustering dendrogram. The fact that members of cluster B do not form a clade in TUD-based comparisons does not invalidate grouping of phage into clusters, but rather serves as a way to highlight phages where TUD and gene or sequence comparisons capture different relationships.

Comparing TUD in a sliding window can highlight regions with dissimilar tetranucleotide composition and identify genomic segments that could have been horizontally transferred. We found self-similar yet genome-different regions at the end of cluster E and L genomes. The new TUD ‘space’ occupied by these segments could be from HGT – a recently transferred genomic section that had not yet ameliorated to the average genome TUD profile. At least for cluster L, we can say that HGT is likely not the cause. Two groups of repetitive sequences at the end of the genome are driving the difference in TUD. However, we found neither repetitive sequences nor a putative transfer candidate for the segment in cluster E. An improvement on our method could potentially detect legitimate HGT events, but we note that the concept of phams ( Hatful *et al.*, 2010) and the computer program Phamerator ( Cresawn *et al.*, 2011) are already efficient for detecting and visualizing these features.

TUD vectors are similar between subcluster B3 phages but different from other members of cluster B. We found that the 4-mer GATC and 6-mer GGATCC were present over four times more than expected in B3 genomes. These sequences are palindromes and part of recognition sites for restriction enzymes, two characteristics of sequences that are typically underrepresented in prokaryotic genomes. GATC and GGATCC are highly used in all sections of B3 genomes, pointing to genome-wide amelioration of usage frequencies.

Oligonucleotide composition methods do not require knowledge of sequence alignment or gene content. They are ideal to compare mycobacteriophage genomes, which lack a common subsequence on which to make alignment-based inference. Alignment-free methods are also valuable when a reference sequence is not available. Recently, methods based on tetranucleotide usage were used to investigate sequences from a gut microbiome and uncovered a population of *Bacteroidales*-like phage that was previously unrepresented in metagenomic sequencing datasets ( Ogilive *et al.*, 2013). Statistics based on oligonucleotide usage are part of a broader class of alignment-free methods. These methods are easy to compute across large datasets: constructing the dendrogram in Supplementary Figure 1 from raw phage sequences takes less than two minutes on a personal laptop. Comparably, creating phylogenetic trees from pairwise global sequence alignment with the Needleman-Wunsch algorithm ( Needleman & Wunsch, 1970) takes over 24 hours on a computing cluster. We envision oligonucleotide usage methods to be used alongside alignment-based techniques. Highlighting large trends and outliers is easy with these methods, but sequence alignment and gene annotation need to be applied to extract biological insights from the data.

### Data and software availability

The genomic sequences of all 663 sequenced mycobacteriophages are publicly available on the website PhagesDB.org as of April 2014. The authors obtained permission to use the data.

### Software access

The code to download mycobacteriophage genome sequences and reproduce our analysis is freely available at https://github.com/bsiranosian/tango_final. Mycobacteriophage genome sequences are available at http://phagesdb.org.

### Latest source code

https://github.com/bsiranosian/tango_final

### Source code as at the time of publication

https://github.com/F1000Research/tango_final

### Archived source code as at the time of publication

http://dx.doi.org/10.5281/zenodo.14609 ( Siranosian *et al.*, 2015b).
