# Supplementary figures and images for: Tetranucleotide usage highlights genomic heterogeneity among mycobacteriophages
Source: F1000Res. 2015 Oct 30;4:36. Originally published 2015 Feb 4. [Version 2] doi: 10.12688/f1000research.6077.2 (PMC4841201; doi:10.12688/f1000research.6077.2)

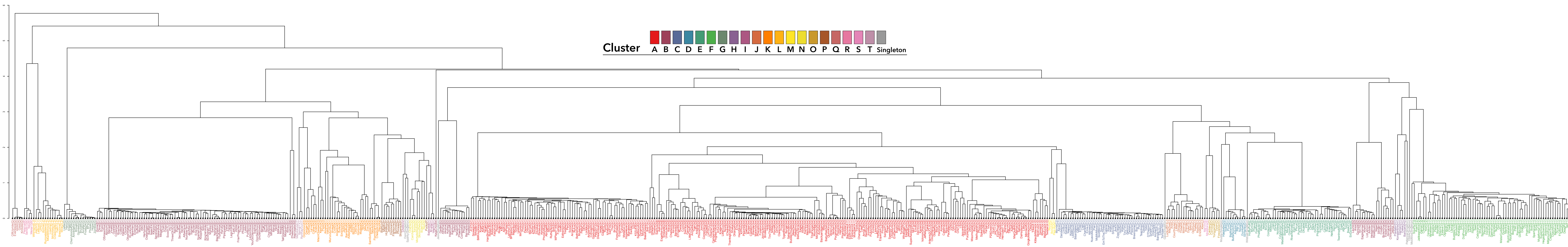

Supplement: Supplementary file 1 [file f1000research-4-7828-s0000.tgz › 5768fb1b-9258-4ce8-838d-1dcde7512dbb.pdf]
