# Supplementary material for: Tetranucleotide usage highlights genomic heterogeneity among mycobacteriophages
Source: F1000Res. 2015 Oct 30;4:36. Originally published 2015 Feb 4. [Version 2] doi: 10.12688/f1000research.6077.2 (PMC4841201; doi:10.12688/f1000research.6077.2)
Supplement: Peer review history

# Peer review: Referee response for version 2

**DOI:** 10.5256/f1000research.7828.r13337

**Published:** 2016-04-20

**Authors:** David Martin

**Affiliations:** 1 Life and Biomedical Sciences Education, School of Life Sciences, University of Dundee, Dundee, UK

The study provides an interesting approach to the evaluation of divergence between the phage genomes. I'm not an expert in this area so come into it with a more general view. I found the revised paper clear and well explained in terms of approach. I agree with the first reviewer that the authors have perhaps been selective in just showing data from a select choice of *k-*mer values. Expanding the results to show the deviation across the full range of *k* tested, even if just in summary, would be interesting, though there would be a disparity between odd and even values of *k *as there are no palindormes with odd *k*.  

A minor issue with regard to the present publication, but which might be worth consideration for future work, is over the TUD metric where the authors compare the observed frequencies to the expected. It is not clear from the study as to the variation one might see in a null model. If TUD is the test statistic of choice, a significance value for the deviation from expected should be deteminable empirically by modelling TUD, e.g.where there is a randomly assigned sequence of nucleotides corresponding to the  genome of the organism. This could be done by shuffling the whole genome, taking a large sliding window and aggregating these scores (with or without shuffling etc.) A discussion of the significance of the deviation from expected (or the lack of appreciation of it) is worth including into the paper.

It is nice to see the distance measures, but without an estimate of the significance of the deviation from expected values, it becomes difficult to assess the significance of the deviation between genomes. It may be the case that using a significance measure as the distance (a Z-score or equivalent) may produce a different clustering.

I have read this submission. I believe that I have an appropriate level of expertise to confirm that it is of an acceptable scientific standard.

# Peer review: Referee response for version 2

**DOI:** 10.5256/f1000research.7828.r11005

**Published:** 2015-11-17

**Authors:** Oliver Bonham-Carter

**Affiliations:** 1 College of Information Science & Technology, School of Interdisciplinary Informatics, University of Nebraska, Omaha, NE, USA

My initial concerns have been addressed.

I have read this submission. I believe that I have an appropriate level of expertise to confirm that it is of an acceptable scientific standard.

## References

[1] Bonham-CarterOSteeleJBastolaD: Alignment-free genetic sequence comparisons: a review of recent approaches by word analysis. *Brief Bioinform*.2014;15(6) : 10.1093/bib/bbt052 890-905 10.1093/bib/bbt052 23904502PMC4296134

# Peer review: Referee response for version 1

**DOI:** 10.5256/f1000research.6506.r7811

**Published:** 2015-03-10

**Authors:** Oliver Bonham-Carter

**Affiliations:** 1 College of Information Science & Technology, School of Interdisciplinary Informatics, University of Nebraska, Omaha, NE, USA

The article is nicely written but sadly, there are elements of discussion which are absent from the paper. If added, the paper's research on mycobacteriophages using alignment-free analysis would have much more support.

- The choice of TUD's as statistics for the alignment-free analysis is not fully explained /justified, nor is there much discussion about what algorithm or method is being employed by the analysis tools of the paper. Are TUD's frequencies? How do these software tools work?
- An simple example of how to calculate a TUD and apply it to a method is necessary to completely understand what they are and to see how they are different from any other motif frequency calculation applied to some other method.
- The assumptions of the methods are not discussed. Many methods from information theory, statistics and other kinds of mathematics require that the input data meets specific requirements (is normal, has a certain distribution, is a frequency, etc.). From the discussion in this paper, the function of analysis tool (the exact algorithm or method) is never clear and so we cannot be sure that the calculations from this work, as applied to these tools, is appropriate. For instance, many tools in information theory require that frequencies be used for their analysis. These frequencies must pass basic rules to be called as such (i.e., found on the scale of 0 to 1, all frequencies must sum to 1, 0 = false, 1 = true). This discussion is not mentioned and if it were, then the choice to used TUDs could be easily integrated into this discussion.
- The manuscript mentioned that k-mers in the range of two to seven were calculated (Methods Section). Where are the results for all these other values of k={2, 3, 5 and 7} which were not the k={4 and 6} results of the article?
- Although other sizes of motifs where apparently used in the analysis, the manuscript focuses on the length-4 motifs. The choice of k=4 for the size of motifs to study is not a very interesting statistic since the probability of a particular length-4 motif showing up randomly in a sequence not very high (1/(4^4) = 1/256). Given that the frequency of mutations, and all the evolutionary time during which to make changes to a sequence, these length=4 similar motifs are likely to randomly turn-up.
- The authors should consider using the occurrence of motifs which are at least seven since these frequencies begin to become less randomly placed. Length-4 words are already common in many many bacteria as restriction sites for restriction enzymes. The authors will also find that there are restriction sites of length-6 for the same purpose and so they will have to remove all restriction enzyme palindromes from their sets of k=4 or 6 sized motifs if they cannot continue with a longer motif length. However, if they are determining the level of conservation between organisms, then having longer motifs should not hurt their results.

Once these issues are addressed, the manuscript will be much stronger.

I have read this submission. I believe that I have an appropriate level of expertise to state that I do not consider it to be of an acceptable scientific standard, for reasons outlined above.

# Response

**Published:** 2015-10-23

**Authors:** Benjamin Siranosian

­Thank you for reviewing the manuscript. I have considered the points you raised, and responded in order below. Changes to the manuscript are noted.

1. The usage deviation-based statistics chosen for this paper are similar to those based on the composition vector of a sequence ( Bonham-Carter *et al*., 2013). Usage deviation (tetranucleotide usage deviation, TUD, in the case of k=4) is a vector of the counts of the possible k-mers, normalized to the expected counts in a randomized genome with the same nucleotide composition. I have made additions to the methods section and included a new figure that makes the calculation of usage deviation more clear. The software tools used to perform these calculations have a description at the github page linked in the paper.
2. I have added an example in the methods section that shows how to calculate TUD for a small sequence. Although this example outlines the method, the results are not very informative. The expected number of any 4-mer is very small in a short sequence, resulting in high TUD values for any 4-mers that do occur.
3. We do not make any assumptions about the input data when calculating usage deviation or performing statistics in the paper.
4. I showed trees constructed from other values of k in Figure 2. The relationships between phage genomes were consistent regardless of the value chosen for k. Other analyses mirrored this result, so we proceed exclusively with k={4, 6}.
5. I agree that length-4 motifs are not interesting to study in isolation. Usage deviation, where values represent deviations from expected frequencies, overcome this point. Single occurrences or counts of any 4-mer are uninteresting. Only when counts are normalized and compared in aggregate do the trends that observed in the paper become meaningful.
6. 7-mers would be less randomly placed in the phage genomes analyzed. Similar to the point above, however, the occurrences of singular k-mers are not considered. As k increases, the resulting usage deviation vectors become sparse. Up to 43% of the (4^7=16384) 7-mers are absent from individual genome sequences, and no 7-mer occurs at least once in every genome analyzed. The sparse nature of the data for 7-mers would not be well-suited to some of the analyses presented in this paper (PCA, searching for horizontally transferred segments).
7. I acknowledge that many 4-mers and 6-mers are restriction sites. In fact, this makes the substrings more interesting. B3 mycobacteriophages have 4 times the expected usage of GATC, a restriction site in some bacteria. Biological sense dictates restriction sites would occur infrequently, but the results say the opposite. I do not feel it is necessary to remove restriction sites before the analysis, and doing so would be somewhat arbitrary. The set of restriction sites in mycobacteria species is not entirely characterized, and the host range for each mycobacteriophage has not been studied.

We hope you find the answers to the points you raised and the revisions to the paper acceptable.

**References:**

Bonham-Carter, O., Steele, J. & Bastola, D. Alignment-free genetic sequence comparisons: a review of recent approaches by word analysis. *Brief Bioinform* **15,** 890–905 (2014).
